# Molecular characterization of exonic rearrangements and frame shifts in the *dystrophin* gene in Duchenne muscular dystrophy patients in a Saudi community

**DOI:** 10.1186/s40246-018-0152-8

**Published:** 2018-04-10

**Authors:** Nasser A. Elhawary, Essam H. Jiffri, Samira Jambi, Ahmad H. Mufti, Anas Dannoun, Hassan Kordi, Asim Khogeer, Osama H. Jiffri, Abdelrahman N. Elhawary, Mohammed T. Tayeb

**Affiliations:** 1 Department of Medical Genetics, Medicine College, Umm Al-Qura University, P.O. Box 57543, Mecca, 21955 Saudi Arabia; 2 Department of Molecular Genetics, Faculty of Medicine, Ain Shams University, Cairo, 11566 Egypt; 3 Department of Medical Laboratory Technology, Faculty of Applied Medical Sciences, King Abdul-Aziz University, Jeddah, Saudi Arabia; 4 Department of Pediatrics, Al Hada Military Hospital, Al Hada, Saudi Arabia; 5 Department of Plan and Research, General Directorate of Health Affairs, Mecca Region, Ministry of Health, Mecca, Saudi Arabia; 6 Department of Pediatrics, Faculty of Medicine, Cairo University, Giza, Egypt

**Keywords:** Duchenne muscular dystrophy, *Dystrophin* gene, Large rearrangements, Frame shift, MLPA, Saudi community

## Abstract

**Background:**

In individuals with Duchenne muscular dystrophy (DMD), exon skipping treatment to restore a wild-type phenotype or correct the frame shift of the mRNA transcript of the *dystrophin* (*DMD*) gene are mutation-specific. To explore the molecular characterization of *DMD* rearrangements and predict the reading frame, we simultaneously screened all 79 *DMD* gene exons of 45 unrelated male DMD patients using a multiplex ligation-dependent probe amplification (MLPA) assay for deletion/duplication patterns. Multiplex PCR was used to confirm single deletions detected by the MLPA.

**Results:**

There was an obvious diagnostic delay, with an extremely statistically significant difference between the age at initial symptoms and the age of clinical evaluation of DMD cases (*t* value, 10.3; 95% confidence interval 5.95–8.80, *P* < 0.0001); the mean difference between the two groups was 7.4 years. Overall, we identified 147 intragenic rearrangements: 46.3% deletions and 53.7% duplications. Most of the deletions (92.5%) were between exons 44 and 56, with exon 50 being the most frequently involved (19.1%). Eight new rearrangements, including a mixed deletion/duplication and double duplications, were linked to seven cases with DMD. Of all the cases, 17.8% had duplications with no hot spots. In addition, confirmation of the reading frame hypothesis helped account for new *DMD* rearrangements in this study. We found that 81% of our Saudi patients would potentially benefit from exon skipping, of which 42.9% had a mutation amenable to skipping of exon 51.

**Conclusions:**

Our study could generate considerable data on mutational rearrangements that may promote future experimental therapies in Saudi Arabia.

**Electronic supplementary material:**

The online version of this article (10.1186/s40246-018-0152-8) contains supplementary material, which is available to authorized users.

## Background

Dystrophinopathies are the most common form of muscular dystrophy in childhood. They are caused by mutations in the *dystrophin* gene (*DMD*; OMIM #300377) [1, 2]. Duchenne muscular dystrophy (DMD; OMIM #310200) is a severe form of muscular dystrophy, with an incidence of 1 in 3600–5000 male births [3]. Becker muscular dystrophy (BMD) is a milder form of DMD, with an incidence of 1 in 20,000 male births (BMD; OMIM # 300376) [4].

DMD is characterized by rapidly progressive degeneration and necrosis of the proximal muscles and calf pseudo-hypertrophy. Most *DMD* patients show muscle weakness at age 2 or 3, but it may be seen as early as infancy. Patients commonly lose independent ambulation by the age of 12 and die of dilated cardiomyopathy around the second or third decade. In comparison, patients with BMD exhibit relatively minor pathological symptoms, slower progression, later onset, and longer survival. Patients with an intermediate form of the disease, intermediate muscular dystrophy (IMD), may continue to walk until they are 16 years of age [4, 5].

The *DMD* gene is one of the largest known genes in humans, with 79 exons (approximately 2.4 Mb of genomic DNA) [1] expressing a 427-kDa muscular protein that plays a fundamental role in stabilizing the sarcolemma. It does so by using a complex of glycoproteins associated with dystrophin to link actin filaments within the cytoskeleton and the extracellular matrix. Lack of dystrophin breaks these connections, altering the plasma membrane and finally producing myofiber degeneration and necrosis [6]. Thus, according to the reading frame hypothesis [7], *DMD* mutations that destroy the reading frame result in a truncated, non-functional dystrophin protein associated with a “DMD” phenotype. These mutations frequently generate a premature stop codon that activates nonsense-mediated mRNA decay [8]. On the other hand, mutations that maintain the reading frame can permit semi-functional dystrophin protein and thus give rise to a “BMD” phenotype [4, 9]. Together, these two phenotype-genotype correlations explain more than 92% of all cases [7].

Different types of mutations have been reported in patients with DMD and BMD. These are mainly large rearrangements (deletions in approximately 60–70% of patients and duplications in approximately 7–10%), with the remaining being point mutations (mainly nonsense mutations) and small deletions or insertions [10, 11]. Most gross deletions can be detected by multiplex PCR (mPCR) [12, 13] and are clustered in the proximal and central hot spot regions [14, 15]. Although a large proportion of the duplications were reported many years ago [16, 17], most laboratories do not systematically screen for these rearrangements. Duplication analysis and the determination of at-risk carrier status of the *DMD* gene require quantitative investigation, which is laborious and technically demanding [18, 19]. Previous studies have applied Southern blotting [16, 20], pulsed-field gel electrophoresis, quantitative mPCR [21–23], multiplex amplifiable probe hybridization [24], and comparative genomic hybridization microarray [25].

Given that deletions and duplications of one or more exons are found in the majority (70%) of patients, it is most cost-efficient and labor-efficient to check for these mutations first. A reliable and rapid technique, multiplex ligation-dependent probe amplification (MLPA), has been applied to cover the whole *DMD* gene to detect deletions and duplications and to identify exactly which exons are involved in deletions or duplications [18, 26–29]. This approach reveals whether a given exon is present and allows the copy number of each exon to be calculated by comparing relative peak heights. MLPA can detect both deletions and duplications in patients as well as in female carriers. Compared to array comparative genomic hybridization, MLPA is a low-cost and technically uncomplicated method.

Although several studies have investigated exonic deletions in different populations [11, 30–36], it is unknown where these deletions occur in the Saudi population. Although a few studies have described the molecular diagnosis of DMD in Saudi patients, the large deletions associated with disease were examined in only some exons, and the studies were limited by small sample sizes [37, 38]. Molecular characterization of the large *DMD* gene has been proposed to address large intragenic rearrangements in the whole exons of the *DMD* gene using an MLPA strategy covering nearly 75% of whole gene mutations. Thus, accurate molecular diagnosis may provide information on eligibility for mutation-specific treatments. A plausible frame shift hypothesis suggests how one might reduce disease severity via exon skipping, for example, by correcting the fidelity of the translational reading frame with large *DMD* deletions or restoring the wild type with large *DMD* duplications.

To our knowledge, the present study is the first study using the MLPA strategy to identify genotype-phenotype correlations in *DMD* patients in a Saudi community. Our results will add valuable data on de novo mutations in this population and to the databases of different DMD web pages as well.

## Methods

### Ethics statement and participants

All participants were enrolled under a protocol approved by the Institutional Biomedical Ethics Committee at Umm Al-Qura University (ref. #HAPO-02-K-012). Parents of all participants gave written consent after being informed about the aim of the study.

The study included 45 unrelated male patients with DMD selected from 65 families from the western region of the Kingdom of Saudi Arabia (KSA), including Jeddah, Mecca, Taif, and Hada. Twenty additional eligible male patients did not enroll because their parents refused to share their clinical data, their clinical profiles were incomplete, or their creatine phosphokinase assessments were missing. For each patient included in the study, a clinical data sheet was recorded in the database of the Molecular Genetics Laboratory in the Department of Medical Genetics at Umm Al-Qura University. Clinical information was independent of any molecular DNA data for the *DMD* gene or its protein. *Dystrophin* probands were diagnosed by clinical geneticists or pediatricians based on strict criteria including a clinical presentation expected for DMD, family history of X-linked muscular dystrophy, or muscle biopsy with a dystrophin analysis performed using immunohistochemistry. Clinical diagnosis of dystrophin probands included age at onset, age at clinical evaluation, calf pseudohypertrophy, age at wheelchair confinement, cardiac function, and motor function. A histopathological study was performed before molecular DNA analysis if muscle biopsies were available. To avoid bias, we included only one case for each family. We categorized patients according to age at loss of ambulation: DMD ≤ 12 years, IMD 12–16 years, and BMD > 16 years. Cases with a family history of autosomal recessive inheritance or with normal dystrophin protein were excluded.

### DNA isolation

Genomic DNA was isolated from buccal cells using the Oragene DNA-OGR-575 kit (DNA Genotek Inc., Ottawa, ON, Canada) according to the manufacturer’s protocol with some modifications. Briefly, the full buccal cells were collected within 30 min, and the Oragene tube was capped immediately. The cells were incubated with the OGR-lysis buffer in a water bath at 53 °C to release the DNA, which was then precipitated by ethanol and dissolved in elution buffer [39].

### Multiplex polymerase chain reaction

The genomic DNA of all *DMD* patients was subjected to multiplex PCR (mPCR) to screen for *DMD* deletions using 15 primer sets (Additional file 1: Table S1). The oligonucleotides included flanking sequences of exons 4, 8, 12, 17, 19, 44, 45, 48, and 51 [12] and of exons 6, 13, 47, 50, 52, and 60 [13]. We made some modifications to Chamberlain’s mPCR set by not adding dimethylsulfoxide, which could result in a lower PCR yield. However, PCR cycling was programmed as initial denaturing at 95 °C for 6 min (1 round), then 94 °C for 30 s, annealing at 53 °C for 30 s, 65 °C for 4 min (repeated for 23 rounds), and final elongation at 65 °C for 7 min [12]. Hot-start mPCR was performed using Beggs’ PCR program [13]: 95 °C for 6 min (1 round) and 25 subsequent cycles including DNA denaturing at 95 °C for 30 s, annealing at 56 °C for 1 min, and elongation at 68 °C for 4 min. Amplification reactions were carried out on thermal cycler Engine Dyad (Bio-Rad Laboratories Inc., Hercules, CA). PCR products (10–15 μl) were separated on 3% NuSieve agarose (BMA Bioproducts, Rockland, ME). The gels were viewed using the Gel Documentation and Analysis System (G-Box, SynGene, Frederick, MD, USA).

### Multiplex ligation-dependent probe amplification

We analyzed all DMD cases for large deletions and large duplications using MLPA SALSA P034/P035 DMD kits (http://www.mrc-holland.com) following the manufacturer’s instructions. In brief, denaturation, hybridization, ligation, and amplification steps were performed on a DNA Engine Dyad thermal cycler (Bio-Rad Laboratories Inc., Hercules, CA). Finally, PCR amplification was performed using SALSA MLPA PCR primers labeled with the FAM dye. A mixture of 0.7 μl of PCR product, 0.2 μl of 600 LIZ GS size-standard, and 9.0 μl of Hi-Di formamide was incubated for 3 min at 86 °C and cooled at 4 °C for 2 min. The MLPA product mix was separated on a POP7 polymer (Applied Biosystems Inc., Life Technologies, Foster City, CA) at 60 °C with the setting of 1.6 kV for injection voltage, 18 s for injection time, 15 kV for run voltage, and 1800 s for run time.

### Data analysis

The raw data were analyzed using GeneMapper Software 5 (Applied Biosystems Inc., Life Technologies, Foster City, CA). The DNA of cases with single-exon deletions were re-examined using conventional PCR. Initial analysis was performed with the naked eye to look for missed exon-specific peaks. For the remaining samples, the peak height of each exon was divided by the two nearest control peaks. The median ratio across all samples for each peak was calculated and used as a reference for one copy. For the sake of accuracy, any normalized ratio below 0.3 was considered a possible deletion. A duplication was considered if a normalized ratio was 1.8–2.0. If any single-exon deletion was identified, conventional PCR amplification was carried out to validate this deletion using primer sets and PCR conditions given in the Leiden Muscular Dystrophy pages (http://www.dmd.nl).

### Databases and confirming mutations for the DMD gene

We checked all mutations recorded in this study according to available databases established by the Leiden Muscular Dystrophy pages (http://www.dmd.nl) [40], the Leiden Open Variation Database 3.0 (http://www.lovd.nl/3.0/home) [41], and UMD-DMD (http://www.umd.be/DMD/) [31, 32]. Databases for exon skipping to restore the DMD reading frame were found on sites developed by Leiden University Medical Center (http://www.exonskipping.nl/?s=exon+skipping&submit=Go) and CureDuchenne (https://www.cureduchenne.org/cure/edystrophin/).

#### Statistical analysis

Hardy-Weinberg equilibrium (HWE) deviation was examined for X-linked *DMD* cases in this study using the Online Encyclopedia for Genetic Epidemiology studies software (http://www.oege.org/software/hwe-mr-calc.shtml). We used the G*Power Software (http://www.psycho.uni-duesseldorf.de/abteilungen/aap/gpower3/download-and-register/) to estimate power analysis to determine adequate sample sizes to achieve an 80% power for *t* testing of point biserial model. “*Priori*” sample size and “*post hoc*” power estimations were tested knowing our DMD sample size, a probability of *α* = 0.05, and the effect size index “*r*” (the absolute value of the correlation coefficient in the population, 0 < “*r*” < 1). We used paired *t* test analysis to compare the significant difference between the age at onset and the age of clinical evaluation for each *DMD* case. A two-sided *P* value less than 0.05 was considered to indicate statistical significance and 95% confidence interval (CI) for all analyses.

## Results

### Clinical profile

**Fig. 1:**
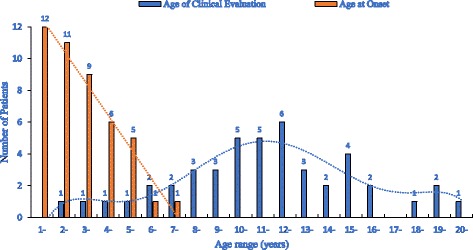
The age at onset and the age of clinical evaluation of DMD patients in this study. The analysis of DMD cases showed an apparent diagnostic delay

Among 45 unrelated patients, 21 were diagnosed with DMD, 10 with IMD, and 5 with BMD. The unassigned patients were defined as not determined (ND), as they were too young to permit a definitive diagnosis (*n* = 9). The median age at onset was 3.5 years (range 1.0–7.0 years), while the median presenting age was 11.5 years (1.5–20 years) (Fig. 1). Most of the patients reported initial symptoms between 1 and 3 years of age (71.1%, 32/45), followed by those reporting symptoms at 4–5 years (24.4%, 11/45). The age at clinical evaluation was most frequently between 10 and 12 years (35.6%, 16/45). We found an extremely statistically significant difference between the age at initial symptoms and the age at clinical evaluation of DMD cases (*t* value, 10.3; 95% CI 5.95–8.8, *P* < 0.0001). The mean difference in age between the two groups was 7.4 years.

### Hardy-Weinberg equilibrium

All affected males were in HWE at the *DMD* gene deletions/duplications (*χ*^2^ = 1.00, *P =* 0.317), where the heterozygotes were absent in such X-linked recessive mode of inheritance.

### Large-scale rearrangements

Using mPCR, we identified 55 large deletions in the 45 unrelated *DMD* patients. MLPA detected 147 intragenic rearrangements, 68 (46.3%) of which were large deletions and 79 (53.7%) of which were large duplications. All deletions identified by mPCR were confirmed by the MLPA-based screening. The utility of MLPA assay for all exons is clear, as 13 (19%) of 68 deletions were detected using MLPA but were not detected by conventional mPCR analysis. The percentage of cases with deletions and duplications were 46.7% (21/45) and 17.8% (8/45), respectively. Table 1 includes the large rearrangements that were identified in the present study and had been previously described.

**Table 1:** Previously described large rearrangements identified in this study and their reading frame shifts

Family no.	Phenotype	Multiplex PCR	MLPA del/dup	Exon(s) del/dup	Codons del/dup	Frame shift	Amino acid change^a^	cDNA^a^
DS-23	DMD	No del	Del 10–11	371	123 ^2/3^	Stop at 323	p.His321PhefsX3	c.961_1331del
DS-1	DMD	Del 19	Del 18–20	454	151 ^1/3^	− 1	p.Arg723Lys874	c.2169_2622del
DS-37	IMD	Del 44	Del 44	148	49 ^1/3^	Stop at 2113	p.Arg2098AsnfsX16	c.6291_6438del
DS-34	ND	Del 44–48	Del 44–48	808	269 ^1/3^	− 1	p.Arg2098Gln2366del	c.6291_7098del
DS-24	DMD	Del 45	Del 45	176	58 ^2/3^	Stop at 2163	p.Glu2147AlafsX17	c.6439_6614del
DS-8	IMD	Del 45–50	Del 45–50	871	290 ^1/3^	Stop at 2155	p.Glu2147LeufsX9	c.6439_7309del
DS-38	IMD	Del 45–52	Del 45–52	1222	407 ^1/3^	Stop at 2168	p.Glu2147LeufsX22	c.6439_7660del
DS-29	DMD	Del 47–50	Del 47–50	547	182 ^1/3^	Stop at 2263	p.Val2257LeufsX7	c.6763_7309del
DS-20	ND	Del 47–50	Del 47–50	547	182 ^1/3^	Stop at 2263	p.Val2257LeufsX7	c.6763_7309del
DS-48	BMD	Del 48	Del 48	186	62	*In-frame*	p.Val2305Gln2366del	c.6913_7098del
DS-30	DMD	Del 50	Del 49–50	211	70 ^1/3^	Stop at 2375	p.Glu2367LeufsX9	c.7099_7309del
DS-31	IMD	Del 50	Del 49–50	211	70 ^1/3^	Stop at 2375	p.Glu2367LeufsX9	c.7099_7309del
DS-36	DMD	Del 50	Del 50	109	36 ^1/3^	Stop at 2409	p.Arg2401LeufsX9	c.7201_7309del
DS-32	IMD	Del 50	Del 50	109	36 ^1/3^	Stop at 2409	p.Arg2401LeufsX9	c.7201_7309del
DS-33	ND	Del 50	Del 50	109	36 ^1/3^	Stop at 2409	p.Arg2401LeufsX9	c.7201_7309del
DS-35	ND	Del 50	Del 50	109	36 ^1/3^	Stop at 2409	p.Arg2401LeufsX9	c.7201_7309del
DS-27	ND	Del 50–52	Del 50–52	460	153 ^1/3^	Stop at 2422	p.Arg2401LeufsX22	c.7201_7660del
DS-12	DMD	Del 51	Del 51	233	77 ^2/3^	Stop at 2469	p.Ser2437CysfsX33	c.7310_7542del
DS-18	DMD	No del	Del 55	190	63 ^1/3^	Stop at 2700	p.Val2677ThrfsX24	c.8028_8217del
DS-11	ND	No del	Dup 50–51	343	114	*In-frame*	p.Arg2401Lys2514dup	c.7201-?_7542 +?dup

*DMD* Duchenne muscular dystrophy, *IMD* intermediate muscular dystrophy, *BMD* Becker muscular dystrophy, *ND* not determined

^a^These data are based on the Leiden Muscular Dystrophy Pages (http://www.dmd.nl/) and the UMD-DMD (http://www.umd.be/DMD/)

### New mutations in the *DMD* gene

We also identified seven previously undescribed large *DMD* rearrangements from eight Saudi cases. These new mutations, based on the *DMD* databases [31, 32, 40, 41], included one mixed rearrangement (del 45–52 + dup 21–23), one large deletion (del 45–56), two large duplications (8–30 and 17–24), and three double duplications (dup 2–4 + dup 18–19, dup 13 + dup 21–24, and dup 56–58 + dup 62–64). These large mutations were from eight (17.8%) of the Saudi patients (Table 2).

**Table 2:** New *DMD* mutational rearrangements identified in this study and their predicted reading frame shifts

Case no.	Phenotype	Multiplex PCR	MLPA del/dup	Exon(s) del/dup	Codons del/dup	Frame shift	Amino acid change^a^
DS-2	DMD	No del	dup 2–4 and dup 18–19	233; 212	77 ^2/3^ 70 ^2/3^	+ 2 + 2	p.Val89MetfsX15 ^b^ p.Lys724Gly795fsX1
DS-14	DMD	No del	dup 8–30	3679	1226 ^1/3^	+ 1	p.Val218K1412fsX
DS-15	ND	No del	dup 8–30	3679	1226 ^1/3^	+ 1	p.Val218K1412fsX
DS-52	BMD	No del	dup 13 and dup 21–24	120 654	40 218	0 0	p.Val495Val535dup p.Asp875Lys1093dup
DS-50	BMD	No del	dup 17–24	1248	428	0	p.Ile665Lys1093dup
DS-53	IMD	del 45–51	del 45–52 and dup 21–23	1222; 540	407 ^1/3^ 180	− 1^c^ 0	p.Glu2147LeufsX22 ^C^ p.Asp875Asn1055dup
DS-25	IMD	del 45–51	del 45–56	1952	650 ^2/3^	− 2	p.Glu2147Ser2798
DS-22	DMD	No del	dup 56–58 and dup 62–64	451 198	150 ^1/3^ 66	+ 1 0	p.Ser2798Lys2891fsX4 p.Ser3056Asp3122

*DMD* Duchenne muscular dystrophy, *IMD* intermediate muscular dystrophy, *BMD* Becker muscular dystrophy, *ND* not determined

^a^Theoretical amino acid change based on the database of the Leiden Muscular Dystrophy Pages (http://www.dmd.nl/)

^b^Previously described duplication at cDNA (c.6439_7660del) showing amino acid change (p.Glu2147LeufsX22) resulting in a termination transcript at codon 2168

^c^Previously described deletion at cDNA (c.32_265dup) showing amino acid change (p.Val89MetfsX15) resulting in a termination transcript at codon 103

### Distribution of rearrangements

**Fig. 2:**
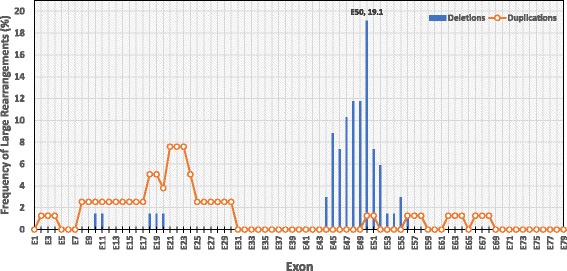
The frequency of large mutational rearrangements for each exon of the *DMD* gene. A region with a high frequency of deletions was found in exons 44–56. No such region of frequency was detected for large duplications

In this study, deletions did not have a random distribution. We found that 92.5% (63/68) of hot spot deletions were linked to exons 44–56 (central region), whereas 7.5% (5/68) of deletions were related to exons 10–20. Exon 50 was most frequently involved in deletions (19.1%, 13/68), followed by exons 48 and 49 (each 11.8%, 8/68). The rate of deletions increased from a minimum in exon 44 to a maximum in exon 50 and then decreased until the 3′ end of the *DMD* gene, with no deletion in exons 57–79 (distal region) (Fig. 2). Moreover, we found that the number of cases with a deletion of *only* one exon was lower than the number with deletions of more than one exon (9/21, 42.8% versus 12/21, 57.1%). About half of the deletions (44.4%) were detected only once, in agreement with the high allelic heterogeneity of the *DMD* gene.

Duplications were distributed in the proximal (68/79, 86.1%), central (5/79, 6.3%), and distal regions (6/79, 7.6%) (Fig. 2). Unlike deletions, duplicated exons were more frequent in the proximal region (26 duplications) than in the central and distal regions (13 duplications). The most frequent duplications were of exons 21, 22, and 23 (7.6%, 6/79 each), followed by exons 18 and 19 (5.1%, 4/79 each). We did not find any duplications in exons 31–49 (central region) or exons 69–79 (distal region) within our cases (Fig. 2). Similar to deletions, 42.5% of exonic duplications (17/40) were observed only once, revealing a considerable heterogeneity of duplications.

### Reading frame shift and phenotype correlation

**Fig. 3:**
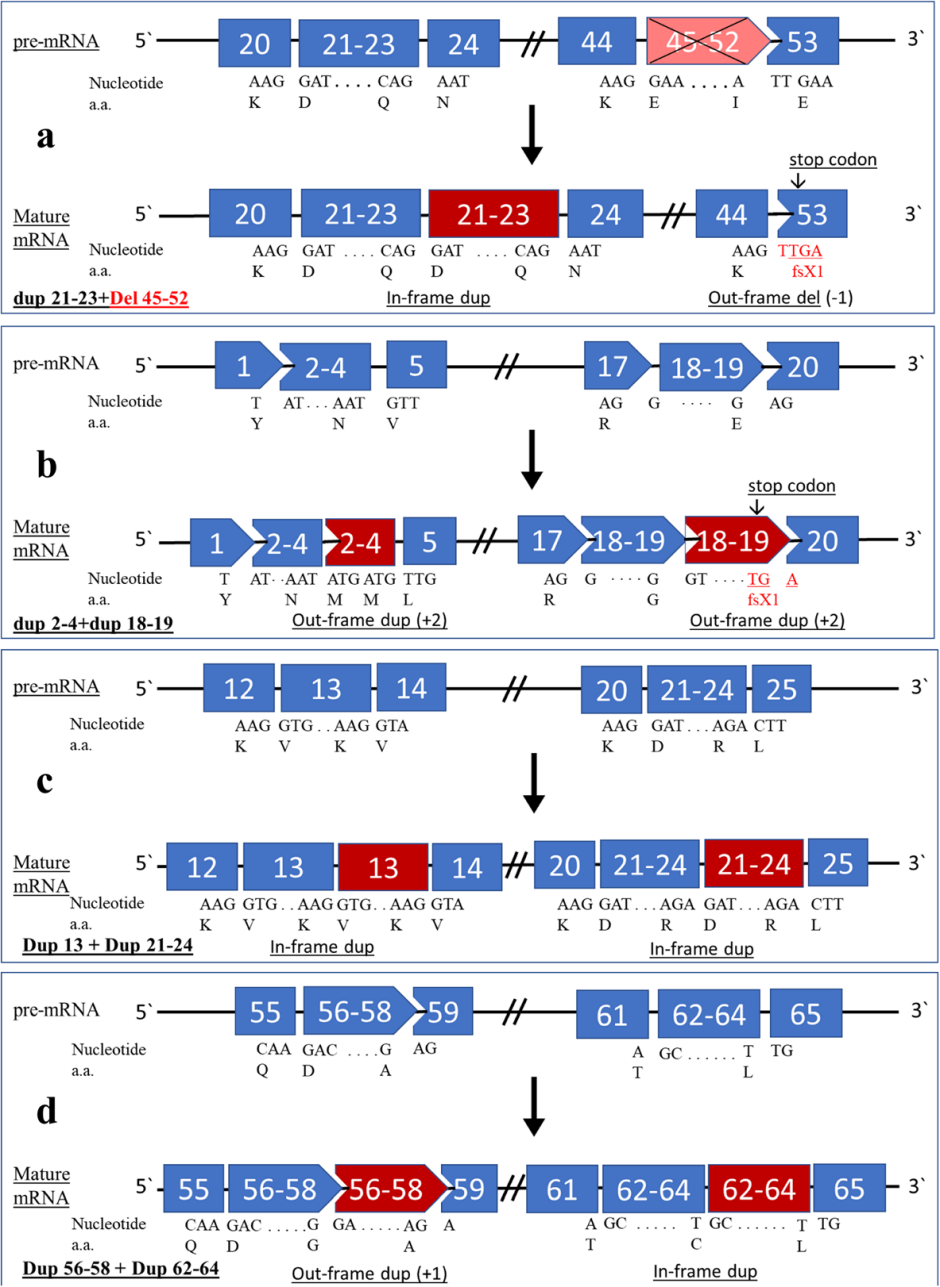
A schematic overview of new complex large rearrangements in the *DMD* gene. **a** The case #DS-53 with an unusual mixed rearrangement (dup 21–23 + del 45–52) leads to an out-of-frame shift giving rise to a severe DMD phenotype. **b** The case #DS2 with a double duplication (dup 2–4 + dup 18–19) results in out-of-frame shifts with a DMD phenotype. **c** The case #DS-52 with two *in-frame* shift due to double duplications (dup 13 + dup 21–24) giving rise to a BMD phenotype. **d** The case #DS-22 showed two double duplications within the mature mRNA giving an out-frame (dup 56–58), in-frame (dup 62–64) mutations giving rise to a DMD phenotype

Gene rearrangements (deletions and duplications) were correlated with clinical phenotypes in 28 unrelated cases: 11 (39.3%) with DMD, 7 (25%) with IMD, 3 (10.7%) with BMD, and 7 (25%) with ND. We also predicted the translational reading frame in 28 DMD cases with rearrangements identified in this study, using the Leiden Muscular Dystrophy pages (http://www.dmd.nl). Applying the reading frame rule revealed consistency with the frame shift rule for 90.9% (10/11) of the individuals with DMD phenotypes and 100% (7/7) of the individuals with IMD phenotypes. Likewise, the *DMD* genes in all cases with BMD phenotypes had in-frame functional effects on the DMD protein (cases #DS-48, #DS-50, and #DS-52) (Tables 1 and 2). All previously described rearrangements we detected gave rise to a stop codon and thus a truncated protein, except for case #DS-48 with a BMD phenotype and case #DS-11 with ND, which gave rise to in-frame predictions (Table 2). Two cases identified in this study (#DS-53 and #DS-22) reflected both in-frame and reading frame shift predictions (Fig. 3). The complex rearrangement of case #DS-53 (del 45–52 + dup 21–23) was associated with an IMD phenotype, with translational reading frame predictions with in-frame and frame shift patterns (Fig. 3). This phenotype may have occurred from the addition of exons 21–23 to the mRNA transcript lessening the damaging effect of del 45–52 on the functional protein. On the contrary, case #DS-22 could not have corrected for the harmful dup 56–58, giving rise to a DMD phenotype (Table 2).

## Discussion

The present study used a facile, reliable, and time-consuming MLPA strategy to identify large rearrangements covering all 79 exons of the *DMD* gene. Our results showed the prevalence of 46.7 and 17.8%, respectively, for large deletions and large duplications in 45 Saudi patients with DMD. Unlike the hot spot deletions in exons 44–56 (92.5%), the hot spot deletions near the 5′ end of the gene were not distinctive, and no large hot spot duplications were found anywhere along the *DMD* gene. The presence of an unusual MLPA pattern in our Saudi sample, including non-contiguous duplications as well as contiguous deletions combined with non-contiguous duplications, suggests complex rearrangements. Our findings regarding double, separate duplications and complex rearrangements are consistent with some previous reports in Serbian and South African patients [18, 42].

Results from MLPA-related studies among different ethnic populations are conflicting in terms of rates of large rearrangements within the *DMD* gene. Studies have found rates of deletions (and duplications) of 71.8–79.0% (16.4–19.8%) in Chinese [29], 79.5% (6.5%) in Indian [43], 60% (10.0%) in Japanese [44], 45.5–71.8% (16.7%) in Korean [35], and 28.2% (20.5%) in Taiwanese [45] populations. When compared with our sample, a Turkish sample has also been shown to have a relatively higher rate of deletions within the *DMD* gene (63.7%) [46], likely because of admixture with other European ethnicities. Rates of *DMD* deletions and duplications in some other Middle Eastern populations are more similar to what we found: Egyptian (51.3% deletions) [47], Iranian (51% deletions) [48], Moroccan (51% deletions) [49], and Syrian (49.0% deletions; 9.8% duplications) [50]. The majority of the reported *DMD* gene mutations in our Saudi data showed translational reading frame shifts (94.4%), while 5.6% of the mutations did not follow the reading frame rule. This latter outcome is relatively consistent with the corresponding values in the TREAT-NMD DMD Global database (7%) [11], the UMD-DMD database (4%) [32], and the Leiden database (9%) [41].

The overall rate of consanguinity in KSA is 57.7%, ranging from 34 to 80.6% [51], with lower rates in Mecca (North Western region) than in Riyadh (Central region) (44.1% versus 62.8%) [51]. This may account for the increased deletions in patients of Riyadh (21/27, 77.8%) when compared with those in our study [37]. During Muslim immigration from the Levant, Africa, in Ancient Islamic times, much intermarriage reinforced gene flow of the *DMD* gene to the Saudi people. This has likely influenced the prevalence of different Mendelian patterns, particularly X-linked types, exemplified by the consistency of data for *DMD* rearrangements between our study and a recent Spanish cohort study (46.1%, 131/284 for deletions and 56/284, 19.7% for duplications) [52].

It is noteworthy that some populations have inherent reproductive barriers that prevent interbreeding, which keeps them at native levels without merging (i.e., cryptic taxa). Other populations may lack inherent reproductive isolation. Therefore, admixture among different geographical populations might increase genetic variations and perhaps create new genotypic combinations within non-isolated (or non-native) populations [53]. Thus, genetic variations among Gulf Arabs and some Middle Eastern individuals (e.g., Barbarians in North Africa, Kurdish, Upper Egyptian) [54, 55] should be handled with caution, as increased consanguinity, extensive reproductive isolation, and admixture with native source populations (e.g., Black Africans, South Eastern Asians, Caucasians) have had substantial roles in gene flow or founder effects in these populations.

In our study, the analysis of *DMD* cases showed an apparent diagnostic delay, as 69.8% of our patients showed their first symptoms at an early age (1–3 years), but 44% of these patients were 9–12 years old at first clinical examination. Other countries have also reported long delays in diagnosis of the disease, with a mean delay between 1.6 and 2.5 years [56, 57]. In south China, the first symptoms occurred by 3 years of age, but the age at clinical evaluation was 6–8 years [36]. Numerous studies have advocated raising public awareness to identify early symptoms in DMD patients [47, 57, 58], as parents are usually the first to notice symptoms, which prompt them to visit a health professional. To further reduce diagnostic delay, creatine phosphokinase (CPK) testing should be emphasized in primary care and performed as a routine test in children’s physical examinations.

Earlier clinical trials reported the safety and biochemical efficacy of intravenous or intramuscular administration of antisense oligonucleotides (20-30 mer) to bring hope to DMD patients with large deletions [59]. Therefore, inducing exon 51 skipping to restore the open reading frame is an attractive therapeutic strategy that can be achieved with splice-switching oligomers. After the US Food and Drug Administration (FDA) accelerated approval of AVI-4658/eteplirsen (Exondys 51; Sarepta Therapeutics Inc., Cambridge, MA, USA), targeting *DMD* exon 51 skipping, *eteplirsen* was approved and introduced in some countries [60–63]. Eteplirsen is useful for patients with amenable *DMD* deletions, ending at exon 50 and starting at exon 52 [64]. To date, eteplirsen has not been approved by the Saudi FDA (https://www.sfda.gov.sa). Hence, numerous efforts have used antisense oligomers to target exon skipping of exon 53 (SRP4053, PRO053), exon 45 (DS-514b, SRP4045), and exon 44 (PRO044) (https://www.clinicaltrials.gov/beta/home) [62, 65]. Based on our data for deletions, exon skipping could eventually apply to 81% (17/21) of DMD cases with large deletions. Among our Saudi patients with *DMD* gene deletions, the exons most frequently skipped were exons 51 (42.9%, 9/21), 53 (14.3%, 3/21), 44 (9.5%, 2/21), 45 (4.8%, 1/21), 43 (4.8%, 1/21), and 50 (4.8%, 1/21). Wein et al. have recently reported the efficiency of exon skipping in the *DMD* gene, with each duplicated exon expressing a wild-type, full-length mRNA [66]. For more than one duplicated exon, several antisense oligomers can be delivered as a cocktail of drugs to skip larger regions of the transcript. Thus, for duplications in exons 45–55, therapeutic skipping can be applied to more than 60% of all DMD patients [67].

Although the power is conventionally utilized for polygenic disorders, the power under different monogenic model of inheritance has not been systematically considered. This issue could be explained because of wide issues, for example, rate of background variation in disease-associated genes, mode of inheritance, extent of penetrance, and locus heterogeneity.

In contrast to dominant model of inheritance, the incomplete penetrance does not hold for the recessive model, and in consequence, much smaller sample sizes are needed under a recessive model, even in the presence of high locus heterogeneity [68]. According to our *priori* sample size estimations at the effect size “*r*” = 0.3 (medium effect), or “*r*” = 0.5 (strong effect), we would need 64 or 21 sample sizes, respectively, to ensure a power detection of 80%. Thus, post hoc analysis using our DMD sample size data in this study (*n* = 45 cases) could achieve the power of 66.7% (*r* = 0.3) and 98.5% (*r* = 0.5).

Pinning down the spectrum of mutations for *DMD* has been difficult because of poor replication of studies. First, when compared with our study, some studies have had populations with admixed ethnicities, conflicted outcomes, or small sample sizes, which lessen the strength of the overall results. Second, various molecular technologies have been utilized to examine *DMD* patients, resulting in a broad range of false-positive or false-negative results regarding rearrangements. Our study mainly used the *DMD* MLPA test, providing a cheap and straightforward DNA-based test that can screen for deletions and duplications and be performed in any DNA laboratory. Third, insufficient communication between clinicians and geneticists, because of difficulty accessing hospitals of interest, may result in underdiagnosis of critical cases. However, precise coordination between clinicians and geneticists may help promote and improve the genetic diagnosis of dystrophinopathies and ameliorate potential therapies in these cases.

## Conclusions

We detected nine previously undescribed exonic rearrangements within the *DMD* gene, including one unusual mixed rearrangement. MLPA or mPCR can be used to define the molecular characteristics of *DMD* rearrangements and hence the effects of the frame shifts on genotype-phenotype correlations in Saudi patients. This information will also be important for future gene therapy targeting exon skipping of the *DMD* gene. Our clinical characteristics revealed a diagnostic delay, suggesting the need for more public awareness about early symptoms of disease. However, CPK testing should also be performed as a routine test in children’s hospitals and in primary care settings. In KSA, molecular testing of DMD patients should be covered by medical insurance, at least once in a lifetime. This single test could lead to genetic diagnosis of more patients. The large deletions and duplications we identified are predictive and intriguing, but the study needs to be replicated in different ethnic populations of the Middle East, as well as in other Saudi governorates. Though the sample size for this study might not have been large enough to explore the *DMD* mutational mechanisms, extensive sequencing analyses will be needed to discover the *DMD* breakpoints at the nucleotide level. Ongoing analyses of whole-exome sequences for Saudi patients with DMD are being carried out to identify the small breakpoints within the *DMD* gene.

## Additional file

Additional file 1: **Table S1.** Oligonucleotide Sequences of 15 multiplex PCR sets and amplification size fragments. (DOCX 17 kb)

## Abbreviations

BMD: Becker muscular dystrophy
CPK: Creatine phosphokinase
DMD: Duchenne muscular dystrophy
FDA: Food and Drug Administration
IMD: Intermediate muscular dystrophy
KSA: Kingdom of Saudi Arabia
MLPA: Multiplex ligation-probe dependent amplification
ND: Not determined

## References

[1] Hoffman EP, Brown RH, Kunkel LM (1987). Dystrophin: the protein product of the Duchenne muscular dystrophy locus. Cell.

[2] Koenig M, Monaco AP, Kunkel LM (1988). The complete sequence of dystrophin predicts a rod-shaped cytoskeletal protein. Cell.

[3] Emery AE (1991). Population frequencies of inherited neuromuscular diseases—a world survey. Neuromuscul Disord.

[4] Bushby K, Finkel R, Birnkrant DJ, Case LE, Clemens PR, Cripe L, Kaul A, Kinnett K, McDonald C, Pandya S (2010). Diagnosis and management of Duchenne muscular dystrophy, part 1: diagnosis, and pharmacological and psychosocial management. Lancet Neurol.

[5] Jarmin S, Kymalainen H, Popplewell L, Dickson G (2014). New developments in the use of gene therapy to treat Duchenne muscular dystrophy. Expert Opin Biol Ther.

[6] Durbeej M, Campbell KP (2002). Muscular dystrophies involving the dystrophin-glycoprotein complex: an overview of current mouse models. Curr Opin Genet Dev.

[7] Monaco AP, Bertelson CJ, Liechti-Gallati S, Moser H, Kunkel LM (1988). An explanation for the phenotypic differences between patients bearing partial deletions of the DMD locus. Genomics.

[8] Hentze MW, Kulozik AE (1999). A perfect message: RNA surveillance and nonsense-mediated decay. Cell.

[9] Muntoni F, Torelli S, Ferlini A (2003). Dystrophin and mutations: one gene, several proteins, multiple phenotypes. Lancet Neurol.

[10] Flanigan KM, Dunn DM, von Niederhausern A, Howard MT, Mendell J, Connolly A, Saunders C, Modrcin A, Dasouki M, Comi GP (2009). DMD Trp3X nonsense mutation associated with a founder effect in North American families with mild Becker muscular dystrophy. Neuromuscul Disord.

[11] Bladen CL, Salgado D, Monges S, Foncuberta ME, Kekou K, Kosma K, Dawkins H, Lamont L, Roy AJ, Chamova T (2015). The TREAT-NMD DMD Global Database: analysis of more than 7,000 Duchenne muscular dystrophy mutations. Hum Mutat.

[12] Chamberlain JS, Gibbs RA, Ranier JE, Nguyen PN, Caskey CT (1988). Deletion screening of the Duchenne muscular dystrophy locus via multiplex DNA amplification. Nucleic Acids Res.

[13] Beggs AH, Koenig M, Boyce FM, Kunkel LM (1990). Detection of 98% of DMD/BMD gene deletions by polymerase chain reaction. Hum Genet.

[14] Forrest SM, Cross GS, Speer A, Gardner-Medwin D, Burn J, Davies KE (1987). Preferential deletion of exons in Duchenne and Becker muscular dystrophies. Nature.

[15] Oudet C, Hanauer A, Clemens P, Caskey T, Mandel JL (1992). Two hot spots of recombination in the DMD gene correlate with the deletion prone regions. Hum Mol Genet.

[16] Den Dunnen JT, Grootscholten PM, Bakker E, Blonden LA, Ginjaar HB, Wapenaar MC, van Paassen HM, van Broeckhoven C, Pearson PL, van Ommen GJ (1989). Topography of the Duchenne muscular dystrophy (DMD) gene: FIGE and cDNA analysis of 194 cases reveals 115 deletions and 13 duplications. Am J Hum Genet.

[17] Hu XY, Ray PN, Murphy EG, Thompson MW, Worton RG (1990). Duplicational mutation at the Duchenne muscular dystrophy locus: its frequency, distribution, origin, and phenotypegenotype correlation. Am J Hum Genet.

[18] Lalic T, Vossen RH, Coffa J, Schouten JP, Guc-Scekic M, Radivojevic D, Djurisic M, Breuning MH, White SJ, den Dunnen JT (2005). Deletion and duplication screening in the DMD gene using MLPA. Eur J Hum Genet.

[19] Elhawary NA, Shawky RM, Elsayed N (2006). High-precision DNA microsatellite genotyping in Duchenne muscular dystrophy families using ion-pair reversed-phase high performance liquid chromatography. Clin Biochem.

[20] Koenig M, Hoffman EP, Bertelson CJ, Monaco AP, Feener C, Kunkel LM (1987). Complete cloning of the Duchenne muscular dystrophy (DMD) cDNA and preliminary genomic organization of the DMD gene in normal and affected individuals. Cell.

[21] Ioannou P, Christopoulos G, Panayides K, Kleanthous M, Middleton L (1992). Detection of Duchenne and Becker muscular dystrophy carriers by quantitative multiplex polymerase chain reaction analysis. Neurol.

[22] Kodaira M, Hiyama K, Karakawa T, Kameo H, Satoh C (1993). Duplication detection in Japanese Duchenne muscular dystrophy patients and identification of carriers with partial gene deletions using pulsed-field gel electrophoresis. Hum Genet.

[23] Yau SC, Bobrow M, Mathew CG, Abbs SJ (1996). Accurate diagnosis of carriers of deletions and duplications in Duchenne/Becker muscular dystrophy by fluorescent dosage analysis. J Med Genet.

[24] White S, Kalf M, Liu Q, Villerius M, Engelsma D, Kriek M, Vollebregt E, Bakker B, van Ommen GJ, Breuning MH (2002). Comprehensive detection of genomic duplications and deletions in the DMD gene, by use of multiplex amplifiable probe hybridization. Am J Hum Genet.

[25] del Gaudio D, Yang Y, Boggs BA, Schmitt ES, Lee JA, Sahoo T, Pham HT, Wiszniewska J, Chinault AC, Beaudet AL (2008). Molecular diagnosis of Duchenne/Becker muscular dystrophy: enhanced detection of dystrophin gene rearrangements by oligonucleotide array-comparative genomic hybridization. Hum Mutat.

[26] Schouten JP, McElgunn CJ, Waaijer R, Zwijnenburg D, Diepvens F, Pals G (2002). Relative quantification of 40 nucleic acid sequences by multiplex ligation-dependent probe amplification. Nucleic Acids Res.

[27] Schwartz M, Duno M (2004). Improved molecular diagnosis of dystrophin gene mutations using the multiplex ligation-dependent probe amplification method. Genet Test.

[28] Janssen B, Hartmann C, Scholz V, Jauch A, Zschocke J (2005). MLPA analysis for the detection of deletions, duplications and complex rearrangements in the dystrophin gene: potential and pitfalls. Neurogenetics.

[29] Chen C, Ma H, Zhang F, Chen L, Xing X, Wang S, Zhang X, Luo Y (2014). Screening of Duchenne muscular dystrophy (DMD) mutations and investigating its mutational mechanism in Chinese patients. PLoS One.

[30] Nobile C, Toffolatti L, Rizzi F, Simionati B, Nigro V, Cardazzo B, Patarnello T, Valle G, Danieli GA (2002). Analysis of 22 deletion breakpoints in dystrophin intron 49. Hum Genet.

[31] Cotton RG, Auerbach AD, Beckmann JS, Blumenfeld OO, Brookes AJ, Brown AF, Carrera P, Cox DW, Gottlieb B, Greenblatt MS (2008). Recommendations for locus-specific databases and their curation. Hum Mutat.

[32] Tuffery-Giraud S, Beroud C, Leturcq F, Yaou RB, Hamroun D, Michel-Calemard L, Moizard MP, Bernard R, Cossee M, Boisseau P (2009). Genotype-phenotype analysis in 2,405 patients with a dystrophinopathy using the UMD-DMD database: a model of nationwide knowledgebase. Hum Mutat.

[33] Mitsui J, Takahashi Y, Goto J, Tomiyama H, Ishikawa S, Yoshino H, Minami N, Smith DI, Lesage S, Aburatani H (2010). Mechanisms of genomic instabilities underlying two common fragile-site-associated loci, PARK2 and DMD, in germ cell and cancer cell lines. Am J Hum Genet.

[34] Ankala A, Kohn JN, Hegde A, Meka A, Ephrem CL, Askree SH, Bhide S, Hegde MR (2012). Aberrant firing of replication origins potentially explains intragenic nonrecurrent rearrangements within genes, including the human DMD gene. Genome Res.

[35] Suh MR, Lee KA, Kim EY, Jung J, Choi WA, Kang SW (2017). Multiplex ligation-dependent probe amplification in X-linked recessive muscular dystrophy in Korean subjects. Yonsei Med J.

[36] Wang DN, Wang ZQ, Yan L, He J, Lin MT, Chen WJ, Wang N (2017). Clinical and mutational characteristics of Duchenne muscular dystrophy patients based on a comprehensive database in South China. Neuromuscul Disord.

[37] Al-Jumah M, Majumdar R, Al-Rajeh S, Chaves-Carballo E, Salih MM, Awada A, Al-Shahwan S, Al-Uthaim S (2002). Deletion mutations in the dystrophin gene of Saudi patients with Duchenne and Becker muscular dystrophy. Saudi Med J.

[38] Tayeb MT (2010). Deletion mutations in Duchenne muscular dystrophy (DMD) in Western Saudi children. Saudi J Biol Sci.

[39] Elhawary NA, Nassir A, Saada H, Dannoun A, Qoqandi O, Alsharif A, Tayeb MT (2017). Combined genetic biomarkers confer susceptibility to risk of urothelial bladder carcinoma in a Saudi population. Dis Markers.

[40] Aartsma-Rus A, Van Deutekom JC, Fokkema IF, Van Ommen GJ, Den Dunnen JT (2006). Entries in the Leiden Duchenne muscular dystrophy mutation database: an overview of mutation types and paradoxical cases that confirm the reading-frame rule. Muscle Nerve.

[41] White SJ, den Dunnen JT (2006). Copy number variation in the genome; the human DMD gene as an example. Cytogenet Genome Res.

[42] Kerr R, Robinson C, Essop FB, Krause A (2013). Genetic testing for Duchenne/Becker muscular dystrophy in Johannesburg. South Africa S Afr Med J.

[43] Manjunath M, Kiran P, Preethish-Kumar V, Nalini A, Singh RJ, Gayathri N (2015). A comparative study of mPCR, MLPA, and muscle biopsy results in a cohort of children with Duchenne muscular dystrophy: a first study. Neurol India.

[44] Okubo M, Minami N, Goto K, Goto Y, Noguchi S, Mitsuhashi S, Nishino I (2016). Genetic diagnosis of Duchenne/Becker muscular dystrophy using next-generation sequencing: validation analysis of DMD mutations. J Hum Genet.

[45] Liang WC, Wang CH, Chou PC, Chen WZ, Jong YJ. The natural history of the patients with Duchenne muscular dystrophy in Taiwan: a medical center experience. Pediatr Neonatol. 2017. 10.1016/j.pedneo.2017.02.004 28903883

[46] Ulgenalp A, Giray O, Bora E, Hizli T, Kurul S, Sagin-Saylam G, Karasoy H, Uran N, Dizdarer G, Tutuncuoglu S (2004). Deletion analysis and clinical correlations in patients with Xp21 linked muscular dystrophy. Turk J Pediatr.

[47] Elhawary NA, Shawky RM, Hashem N (2004). Frameshift deletion mechanisms in Egyptian Duchenne and Becker muscular dystrophy families. Mol Cells.

[48] Nouri N, Fazel-Najafabadi E, Salehi M, Hosseinzadeh M, Behnam M, Ghazavi MR, Sedghi M (2014). Evaluation of multiplex ligation-dependent probe amplification analysis versus multiplex polymerase chain reaction assays in the detection of dystrophin gene rearrangements in an Iranian population subset. Adv Biomed Res.

[49] Sbiti A, El Kerch F, Sefiani A (2002). Analysis of dystrophin gene deletions by multiplex PCR in Moroccan patients. J Biomed Biotechnol.

[50] Madania A, Zarzour H, Jarjour RA, Ghoury I (2010). Combination of conventional multiplex PCR and quantitative real-time PCR detects large rearrangements in the dystrophin gene in 59% of Syrian DMD/BMD patients. Clin Biochem.

[51] el-Hazmi MA, al-Swailem AR, Warsy AS, al-Swailem AM, Sulaimani R, al-Meshari AA (1995). Consanguinity among the Saudi Arabian population. J Med Genet.

[52] Vieitez I, Gallano P, Gonzalez-Quereda L, Borrego S, Marcos I, Millan JM, Jairo T, Prior C, Molano J, Trujillo-Tiebas MJ (2017). Mutational spectrum of Duchenne muscular dystrophy in Spain: study of 284 cases. Neurologia.

[53] Lavergne S, Molofsky J (2007). Increased genetic variation and evolutionary potential drive the success of an invasive grass. Proc Natl Acad Sci U S A.

[54] Rund D, Cohen T, Filon D, Dowling CE, Warren TC, Barak I, Rachmilewitz E, Kazazian HH, Oppenheim A (1991). Evolution of a genetic disease in an ethnic isolate: beta-thalassemia in the Jews of Kurdistan. Proc Natl Acad Sci U S A.

[55] Jiffri EH, Bogari N, Zidan KH, Teama S, Elhawary NA (2010). Molecular updating of β-thalassemia mutations in the upper Egyptian population. Hemoglobin.

[56] Ciafaloni E, Fox DJ, Pandya S, Westfield CP, Puzhankara S, Romitti PA, Mathews KD, Miller TM, Matthews DJ, Miller LA (2009). Delayed diagnosis in duchenne muscular dystrophy: data from the Muscular Dystrophy Surveillance, Tracking, and Research Network (MD STARnet). J Pediatr.

[57] van Ruiten HJ, Straub V, Bushby K, Guglieri M (2014). Improving recognition of Duchenne muscular dystrophy: a retrospective case note review. Arch Dis Child.

[58] Li X, Zhao L, Zhou S, Hu C, Shi Y, Shi W, Li H, Liu F, Wu B, Wang Y (2015). A comprehensive database of Duchenne and Becker muscular dystrophy patients (0−18 years old) in East China. Orphanet J Rare Dis.

[59] Cirak S, Arechavala-Gomeza V, Guglieri M, Feng L, Torelli S, Anthony K, Abbs S, Garralda ME, Bourke J, Wells DJ (2011). Exon skipping and dystrophin restoration in patients with Duchenne muscular dystrophy after systemic phosphorodiamidate morpholino oligomer treatment: an open-label, phase 2, dose-escalation study. Lancet.

[60] Mendell JR, Goemans N, Lowes LP, Alfano LN, Berry K, Shao J, Kaye EM, Mercuri E, Eteplirsen Study G, Telethon Foundation DMDIN (2016). Longitudinal effect of eteplirsen versus historical control on ambulation in Duchenne muscular dystrophy. Ann Neurol.

[61] Shimizu-Motohashi Y, Miyatake S, Komaki H, Takeda S, Aoki Y (2016). Recent advances in innovative therapeutic approaches for Duchenne muscular dystrophy: from discovery to clinical trials. Am J Transl Res.

[62] Lee BL, Nam SH, Lee JH, Ki CS, Lee M, Lee J (2012). Genetic analysis of dystrophin gene for affected male and female carriers with Duchenne/Becker muscular dystrophy in Korea. J Korean Med Sci.

[63] Lim KR, Maruyama R, Yokota T (2017). Eteplirsen in the treatment of Duchenne muscular dystrophy. Drug Des Devel Ther.

[64] van Deutekom JC, van Ommen GJ (2003). Advances in Duchenne muscular dystrophy gene therapy. Nat Rev Genet.

[65] Mah JK (2016). Current and emerging treatment strategies for Duchenne muscular dystrophy. Neuropsychiatr Dis Treat.

[66] Wein N, Vulin A, Findlay AR, Gumienny F, Huang N, Wilton SD, Flanigan KM (2017). Efficient skipping of single exon duplications in DMD patient-derived cell lines using an antisense oligonucleotide approach. J Neuromuscul Dis.

[67] Aoki Y, Yokota T, Nagata T, Nakamura A, Tanihata J, Saito T, Duguez SM, Nagaraju K, Hoffman EP, Partridge T (2012). Bodywide skipping of exons 45-55 in dystrophic mdx52 mice by systemic antisense delivery. Proc Natl Acad Sci U S A.

[68] Guo MH, Dauber A, Lippincott MF, Chan YM, Salem RM, Hirschhorn JN (2016). Determinants of power in gene-based burden testing for monogenic disorders. Am J Hum Genet.

