# Supplementary material for: Molecular characterization of exonic rearrangements and frame shifts in the dystrophin gene in Duchenne muscular dystrophy patients in a Saudi community
Source: Hum Genomics. 2018 Apr 10;12:18. doi: 10.1186/s40246-018-0152-8 (PMC5891934; doi:10.1186/s40246-018-0152-8)
Supplement: Supplementary file 1 — Table S1. Oligonucleotide Sequences of 15 multiplex PCR sets and amplification size fragments. (DOCX 17 kb) [file 40246_2018_152_MOESM1_ESM.docx]

**Table S1.** Oligonucleotide Sequences of 15 multiplex PCR sets, and amplification size fragments

| Exon* | Size (bp) | Forward primer (5`‒3`) | Reversed primer (5`‒3`) |
| --- | --- | --- | --- |
| Exon 45 | 547 | aaacatggaacatccttgtggggac | cattcctattagatctgtcgccctac |
| Exon 48 | 506 | ttgaatacattggttaaatcccaacatg | cctgaataaagtcttccttaccacac |
| Exon 19 | 459 | gatggcaaaagtgttgagaaaaagtc | ttctaccacatcccattttcttcca |
| Exon 17 | 416 | gactttcgatgttgagattactttccc | aagcttgagatgctctcacCTTTTCC |
| Exon 51 | 388 | gaaattggctctttagcttgtgtttc | ggagagtaaagtgattggtggaaaatc |
| Exon 8 | 360 | ggcctcattctcatgttctaattag | gtcctttacacactttacCTGTTGAG |
| Exon 12 | 331 | gatagtgggctttacttacatccttc | gaaagcacgcaacataagatacacct |
| Exon 44 | 268 | cttgatccatatgcttttacctgca | tccatcacccttcagaacctgatct |
| Exon 4 | 196 | ttgtcggtctctctgctggtcagtg | caaagccctcactcaaacatgaagc |
| Exon 50 | 271 | caccaaatggattaagatgttcatgaat | tctctctcacccagtcatcacttcatag |
| Exon 13 | 238 | aataggagtacctgagatgtagcagaaat | ctgacCTTAAGTTGTTCTTCCAAAGCAG |
| Exon 6 | 202 | ccacatgtagGTCAAAAATGTAATGAA | gtctcagtaatcttcttacCTATGACTATGG |
| Exon 47 | 181 | cgttgttgcatttgtctgtttcagTTAC | gtctaacCTTTATCCACTGGAGATTTG |
| Exon 60 | 139 | AGGAGAAATTGCGCCTCTGAAAGAGAACG | CTGCAGAAGCTTCCATCTGGTGTTCAGG |
| Exon 52 | 113 | AATGCAGGATTTGGAACAGAGGCGTCC | TTCGATCCGTAATGATTGTTCTAGCCTC |

Exons 4, 44, 12, 8, 51, 17, 19, 48, and 48 are Chamberlain’s primer set, and exons 52, 60, 47, 6, 13, and 50 [12] are of Beggs’ primers set [13].

* Each exon sequences set is sorted according to their sizes (in base-pairs).
